# Corticosteroids as Selective and Effective Modulators of Glycine Receptors

**DOI:** 10.1021/acschemneuro.3c00287

**Published:** 2023-08-16

**Authors:** Elena I. Solntseva, Julia V. Bukanova, Rodion Kondratenko, Eva Kudova

**Affiliations:** † Functional Synaptology Laboratory, Brain Research Institute, Research Center of Neurology, Moscow 125367, Russia; ‡ Institute of Organic Chemistry and Biochemistry of the Czech Academy of Sciences, Prague 166 10, Czech Republic

**Keywords:** GABA_A_ receptor, glycine receptor, corticosteroids, structure−activity relationship study

## Abstract

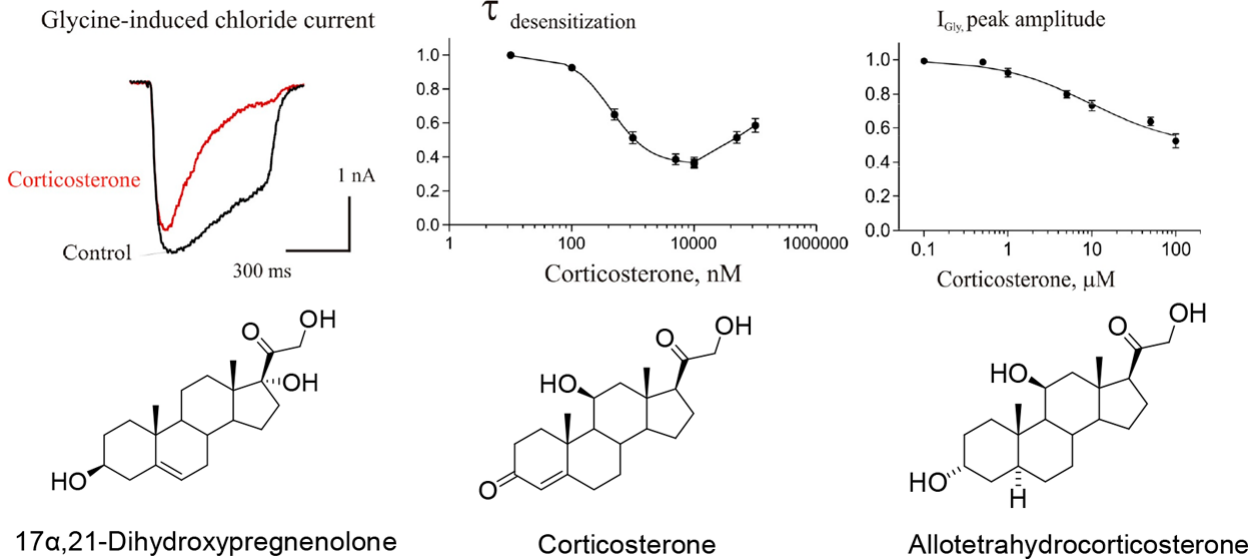

The mechanism of the negative impact of corticosteroids on the induction and progress of mental illness remains unclear. In this work, we studied the effects of corticosteroids on the activity of neuronal glycine receptors (GlyR) and GABA-A receptors (GABA_A_R) by measuring the chloride current induced by the application of GABA (2 or 5 μM) to isolated cerebellar Purkinje cells (*I*_GABA_) and by the application of glycine (100 μM) to pyramidal neurons of the rat hippocampus (*I*_Gly_). It was found that corticosterone, 5α-dihydrodeoxycorticosterone, allotetrahydrocorticosterone, cortisol, and 17α,21-dihydroxypregnenolone were able to accelerate the desensitization of the *I*_Gly_ at physiological concentrations (IC_50_ values varying from 0.39 to 0.72 μM). Next, cortisone, 11-deoxycortisol, 11-deoxycorticosterone, 5β-dihydrodeoxycorticosterone, and tetrahydrocorticosterone accelerated the desensitization of *I*_Gly_ with IC_50_ values varying from 10.3 to 15.2 μM. Allotetrahydrocorticosterone and tetrahydrocorticosterone potentiated the *I*_GABA_ albeit with high EC_50_ values (18–23 μM). The rest of the steroids had no effect on *I*_GABA_ in the range of concentrations of 1–100 μM. Finally, our study has suggested a structural relationship of the 3β-hydroxyl group/3-oxo group with the selective modulatory activity on GlyRs in contrast to the 3α-hydroxyl group that is pivotal for GABA_A_Rs. In summary, our results suggest that increased GlyR desensitization by corticosteroids may contribute to brain dysfunction under chronic stress and identify corticosteroids for further development as selective modulators of GlyRs.

## Introduction

Corticosteroids (cortisol, cortisone, corticosterone, and deoxycorticosterone) are called stress hormones. They are produced during stress by adrenal glands upon activation by adrenocorticotropin, release of which is under the control of the hypothalamic–pituitary–adrenal axis (HPA axis).^1−3^ Acute stress can have a positive effect on behavioral strategies, increasing activity, enhancing blood glucose levels, and thereby promoting adaptation. However, chronic stress reduces activity and often exacerbates the manifestation of the disease.^4^ The relationship between cortisol levels and symptoms of mental diseases has been intensively investigated as chronic stress is a high-risk factor for many psychiatric disorders.^1^ For example, cortisol was identified as a potential predictor for major depressive disorder,^5^ a risk factor for depression in adolescence,^6^ and a known proconvulsant.^7^ It is also proposed to be associated with the progression and severity of multiple sclerosis^8^ and Parkinson’s disease.^9^ In animal experiments, exposure to prenatal stress has been shown to lead to an increase in peripheral corticosterone levels and long-lasting neurobiological and behavioral consequences for the offspring, which may enhance the susceptibility to mental disorders.^10^ Mechanisms of the negative impact of corticosteroids on the induction or progress of mental illness are not clear and remain to be investigated.

**Figure 1:**
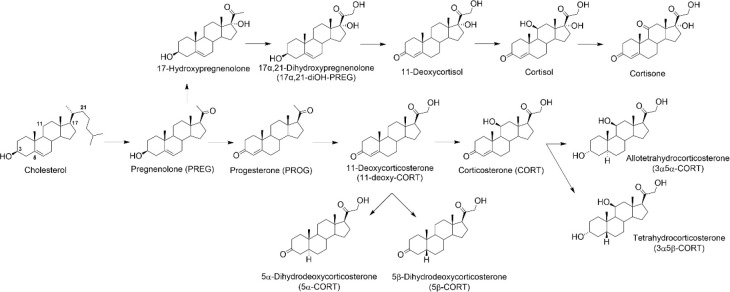
Biosynthesis and metabolism of tested corticosteroids and their neurosteroidal analogues.

The inhibitory GABA system plays an important role in the mechanisms of the body’s response to stress. Acute and chronic stress has been shown to alter GABA neurotransmission in different ways.^7^ In acute stress, an increase in GABA neurotransmission occurs due to the release of neurosteroids, activators of GABA-A receptors (GABA_A_R), into the plasma and brain. In contrast, chronic stress creates a sustained state of neurosteroid deficiency which causes reduced GABA inhibition in the brain.^7^ Neurosteroids, such as allopregnanolone and tetrahydrodeoxycorticosterone (THDOC) have been shown to be released during acute stress from central and adrenal sources and to enhance GABA neurotransmission,^11−14^ which provides neuroprotective and anticonvulsant effects.^3,7,13^ It should be noted that our knowledge of the effect of corticosteroids on GABA_A_R is incomplete. A systematic study targeting effects of corticosteroids and their neurosteroidal metabolites on GABA_A_R has not yet been published. In this article, we have described the effects of 10 corticosteroids and their metabolites on the GABA-activated chloride current in isolated rat Purkinje cells (Figure 1). The tested group includes cortisol, corticosterone (CORT), cortisone, 17α,21-dihydroxypregnenolone (17α,21-diOH-PREG), 5α-dihydrodeoxycorticosterone (5α-CORT), 5β-dihydrodeoxycorticosterone (5β-CORT), 11-deoxycorticosterone (11-deoxy-CORT), 11-deoxycortisol, allotetrahydrocorticosterone (3α5α-CORT), and tetrahydrocorticosterone (3α5β-CORT). A common structural feature of the tested steroids is the presence of a hydroxyl group at the C-21 of the steroid skeleton.

Next, this study investigated the effect of corticosteroids on neuronal glycine receptors (GlyRs). GlyRs are pentameric assemblages, combinations of five types of subunits (α1−α4 and β), which may be α-homopentamers or α/β-heteropentamers.^15^ GlyRs, along with GABA_A_Rs, perform an inhibitory function in the central nervous system.^16^ The disruption of GlyR activity contributes to such brain pathology as hyperekplexia (startle response), autism spectrum disorders, chronic pain, and epilepsy (for review, see ref (17)). GlyRs are a target for a variety of psychoactive drugs since they possess multiple sites for allosteric modulation by structurally diverse molecules: alcohol, cannabinoids, tropeines, anesthetics, and neurosteroids (NSs) (for review, see refs (17 and 18)). The effects of NSs with pregnane and androstane structure on glycine-induced chloride current (*I*_Gly_) were studied on a culture of rat spinal neurons^19−21^ and on recombinant GlyRs expressed in *Xenopus* oocytes.^22,23^ Our recent studies described the effect of NSs on *I*_Gly_ in isolated rat hippocampal pyramidal neurons. Using this model, we have shown that NSs with pregnane, androstane, and androstene structures decrease *I*_Gly_ by accelerating desensitization and reducing peak amplitude.^24−26^ The effects of corticosteroids on *I*_Gly_ have not yet been investigated. In the present work, we studied the effect of ten corticosteroids and their derivatives on *I*_Gly_ in isolated rat hippocampal pyramidal neurons. The results obtained may be useful for understanding the mechanisms of the physiological effects of corticosteroids.

## Results

### Effect of Corticosteroids on *I*_GABA_ and *I*_Gly_

We have previously shown that rat cerebellum Purkinje cells are a convenient model for studying GABA-activated chloride current (*I*_GABA_), and rat hippocampal pyramidal neurons are convenient for studying glycine-activated chloride current (*I*_Gly_).^26^ In this article, the effects of corticosteroids and their derivatives (Table 1) in a concentration range of 0.01–100 μM on the *I*_GABA_ in isolated rat cerebellar Purkinje cells and on the *I*_Gly_ in rat hippocampal neurons were evaluated. First, the ability of steroids to affect the holding current at the voltage-clamp regime was tested. It was shown that the tested compounds at concentrations of 1–100 μM did not affect the holding current (data not shown). Next, the influence of all of the compounds on *I*_GABA_ and *I*_Gly_ was evaluated. Glycine (100 μM) and GABA (2 or 5 μM) were applied to the neurons through an application pipet during 0.6–1 s, and compounds were added to the same pipet in different concentrations (0.01–100 μM). Our experiments demonstrated that steroids tested affected *I*_GABA_ and *I*_Gly_ in a different manner: at low concentrations (up to 10 μM), they augmented or did not change the peak amplitude of the *I*_GABA_ (*I*_GABA-peak_) but reduced the *I*_Gly_ by decreasing the peak amplitude (*I*_Gly-peak_) and/or accelerating desensitization (Table 1). The latter effect was quantified by the change in the time constant of the *I*_Gly_ decay (*I*_Gly_ τ_des_).

**Table 1:**
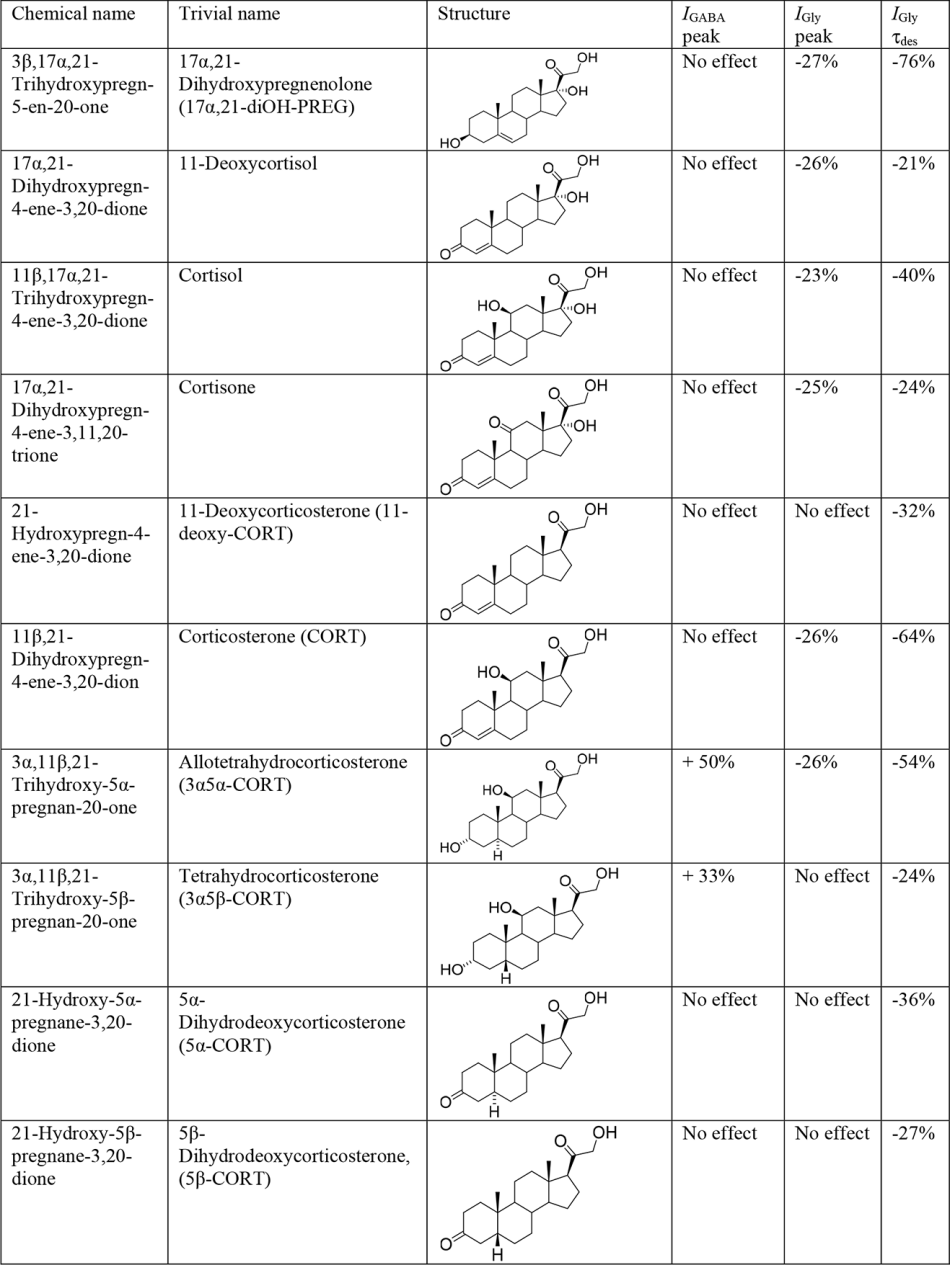
Structure–Activity Relationship Study Overview for Steroids Tested: Their Chemical and Trivial Names, Structures, and Effects at 10 μM on *I*_GABA_ Peak, *I*_Gly_ Peak, and *I*_Gly_ τ_des_

### 3α5α-CORT and 3α5β-CORT Potentiate the *I*_GABA_ in Purkinje Cells from the Cerebellum

**Figure 2:**
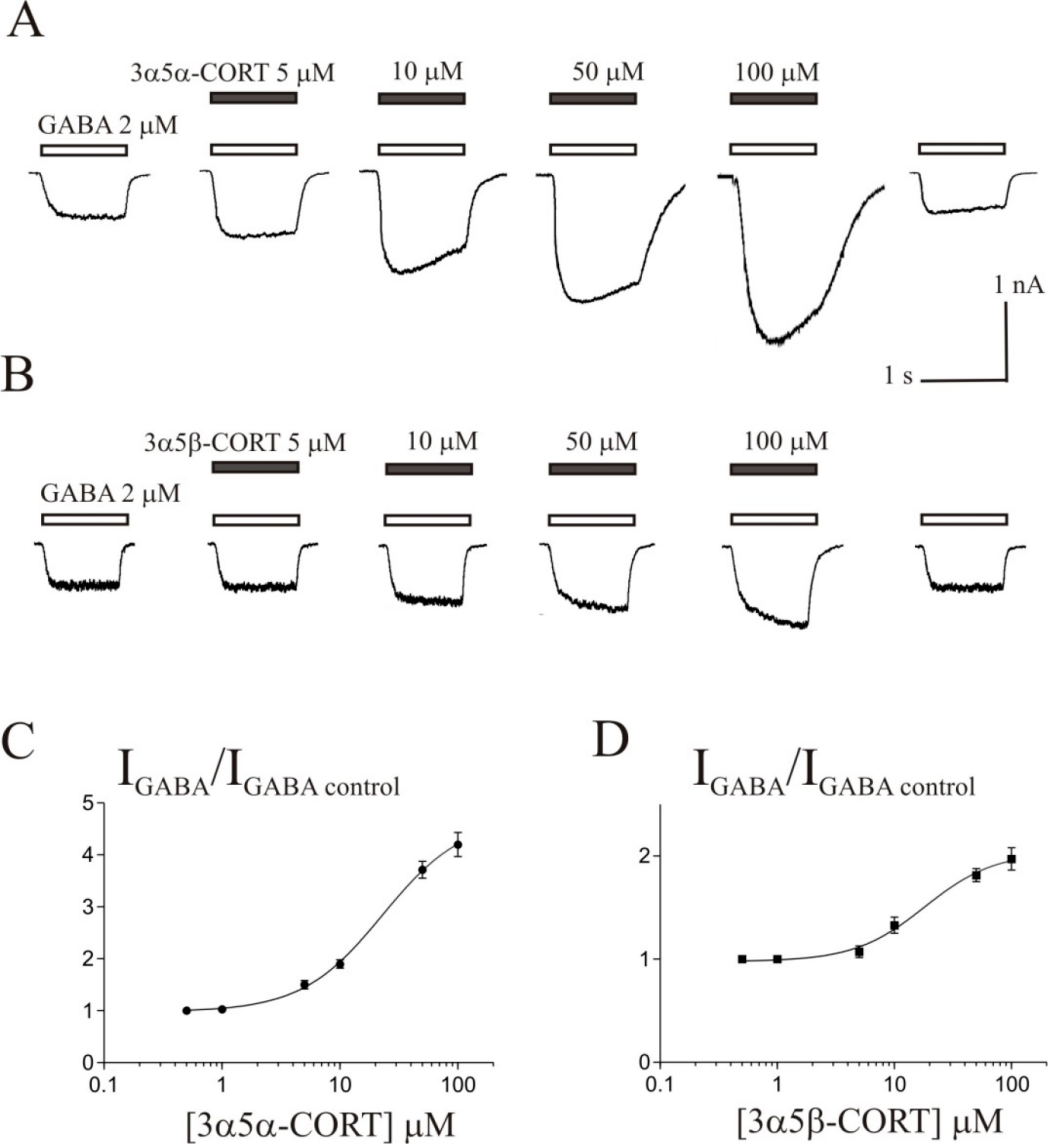
Stimulating effect of allotetrahydrocorticosterone (3α5α-CORT) and tetrahydrocorticosterone (3α5β-CORT) on the GABA-induced chloride current (*I*_GABA_) in Purkinje cells. (A, B) Representative traces of *I*_GABA_ induced by 1 s application of 2 μM GABA, obtained in control and the presence of 5, 10, 50, and 100 μM 3α5α-CORT (A) or 3α5β-CORT (B). (C, D) Concentration dependence of steroid effects on the normalized peak amplitude of *I*_GABA_: 3α5α-CORT (C) and 3α5β-CORT (D). *N* = 7 cells for every point.

The brief application of GABA for 1 s on isolated Purkinje cells evoked *I*_GABA_ with an amplitude dependent on GABA concentration with an EC_50_ value of 6.8 ± 1.0 μM. The specific antagonist of GABA_A_ receptor bicuculline (5 μM) reversibly blocked the current. The average value of the reversal potential of *I*_GABA_ (−9.7 ± 0.8 mV) closely matched the chloride reversal potential calculated for the chloride concentrations used (−9.5 mV). Coapplication of 2 μM GABA (EC_5_) with a different concentration of allotetrahydrocorticosterone (3α5α-CORT) caused potentiation of *I*_GABA_ (Figure 2). The effect was reversible upon washout during 1–2 applications of pure GABA. The threshold concentration of 3α5α-CORT was 5 μM, at which the peak amplitude of the current increased to 150 ± 8% of the control (*p* < 0.001, *N* = 7). A representative effect of 3α5α-CORT on *I*_GABA_ on one cell is shown in Figure 2A. An increase in the steroid concentration up to 100 μM caused a dose-dependent increase in the potentiation effect. The maximum effect (max) was observed at 100 μM 3α5α-CORT and amounted to 420 ± 19% of the control, while the EC_50_ and Hill coefficients were 23 ± 8 and 1.3 ± 0.4, respectively. Figure 2C shows the concentration dependence of the 3α5α-CORT effect on the normalized *I*_GABA_ peak amplitude.

Tetrahydrocorticosterone (3α5β-CORT) also potentiated *I*_GABA_ on Purkinje cells, although to a lesser degree than 3α5α-CORT. The threshold concentration of 3α5β-CORT was 10 μM, at which the peak amplitude of the current increased to 133 ± 8% of the control (*p* < 0.01, *N* = 7). A representative effect of 3α5β-CORT on *I*_GABA_ on one cell is shown in Figure 2B. An increase in the steroid concentration up to 100 μM caused a dose-dependent increase in the potentiation effect. The maximum effect (max) was observed at 100 μM 3α5β-CORT and amounted to 198 ± 11% of the control, while the EC_50_ and Hill coefficient were 18 ± 7 μM and 1.4 ± 0.6, respectively. Figure 2D shows the concentration dependence of the 3α5β-CORT effect on the normalized *I*_GABA_ peak amplitude.

**Figure 3:**
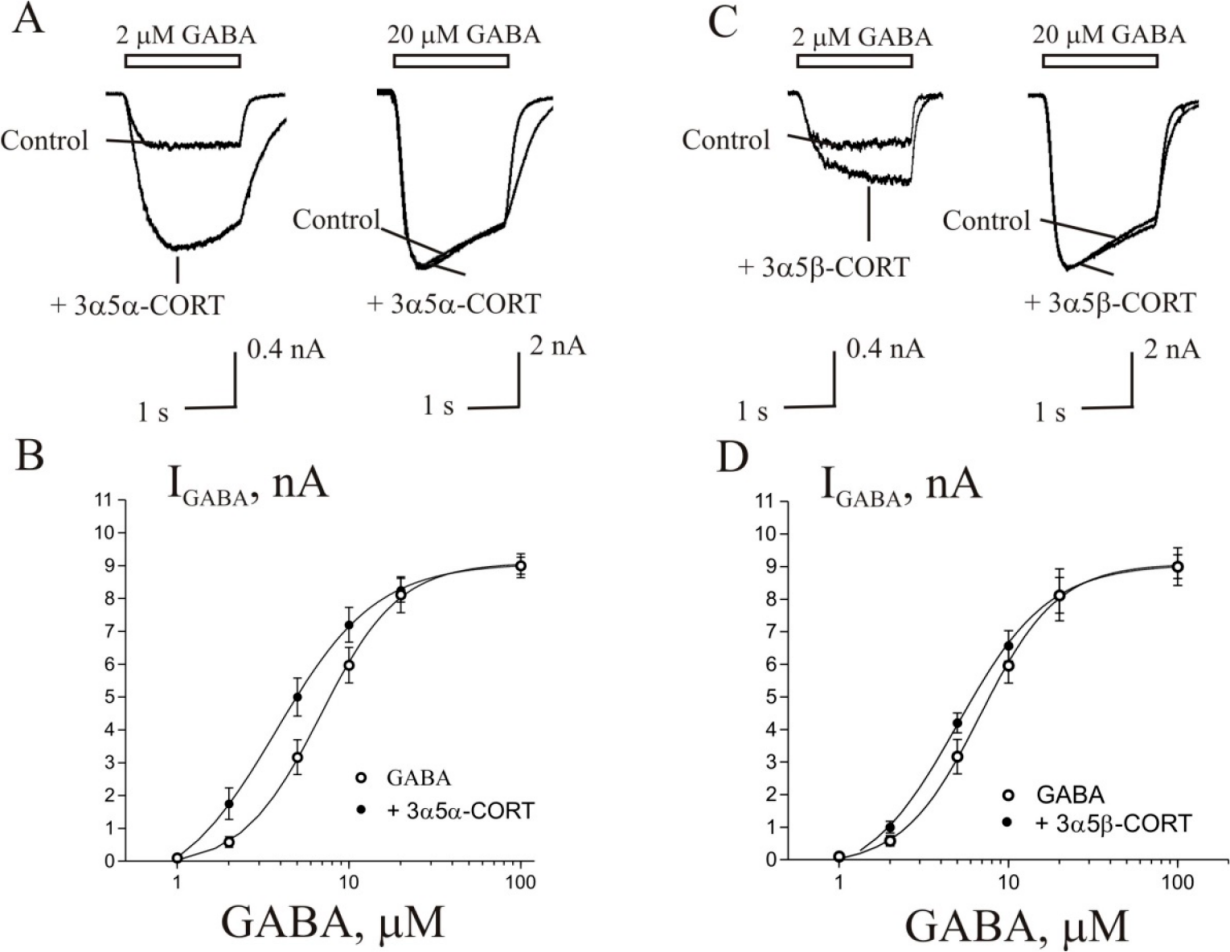
Steroid-stimulating effect on the *I*_GABA_ depends on GABA concentration. (A, C) Representative current traces induced by 2 and 20 μM GABA in control and during coapplication with 50 μM 3α5α-CORT (A) or 50 μM 3α5β-CORT (C). (B, D) Concentration–response curves for GABA were obtained in control (open circles) and in the presence of 50 μM 3α5α-CORT (B) or 50 μM 3α5β-CORT (D) (filled circles). Data points represent the average from six cells.

The influence of GABA concentration on the extent of steroid-induced potentiation was determined by measuring the potentiation by 50 μM 3α5α-CORT and by 50 μM 3α5β-CORT of the *I*_GABA_ evoked by increasing the GABA concentration from 2 to 100 μM (Figure 3). The potentiation was GABA concentration-dependent, being larger at lower concentrations of GABA (Figure 3A,C). The comparison of the concentration–response curve for GABA in control and during coapplication with 3α5α-CORT or 3α5β-CORT shows that the steroid did not change the maximal GABA current but shifted the dose–response curve to the left (Figure 3B,D). Statistical analysis was performed using the paired Student’s *t* test.

The current induced by 2 μM, 5 μM, or 10 μM GABA was enhanced by 3α5α-CORT to 371 ± 16%, 156 ± 15%, and 120 ± 16% of control, correspondingly (*p* < 0.05, *N* = 6). The potentiating effect was not observed at higher GABA concentrations (20 and 100 μM). The EC_50_ value was changed from 6.8 ± 1.0 μM under the control conditions to 3.7 ± 1.4 μM in the presence of 50 μM 3α5α-CORT (*p* < 0.001, *N* = 6). As for 3α5β-CORT, its potentiating effect was manifested only at two concentrations of GABA, 2 and 5 μM (172 ± 18% and 139 ± 16% of control, correspondingly, *p* < 0.05, *N* = 6), and the EC_50_ value of the concentration–response curve decreased to 5.0 ± 1.1 (*p* < 0.01, *N* = 6).

### Corticosteroids without the 3α-OH Group Do Not Cause Potentiation of *I*_GABA_

**Figure 4:**
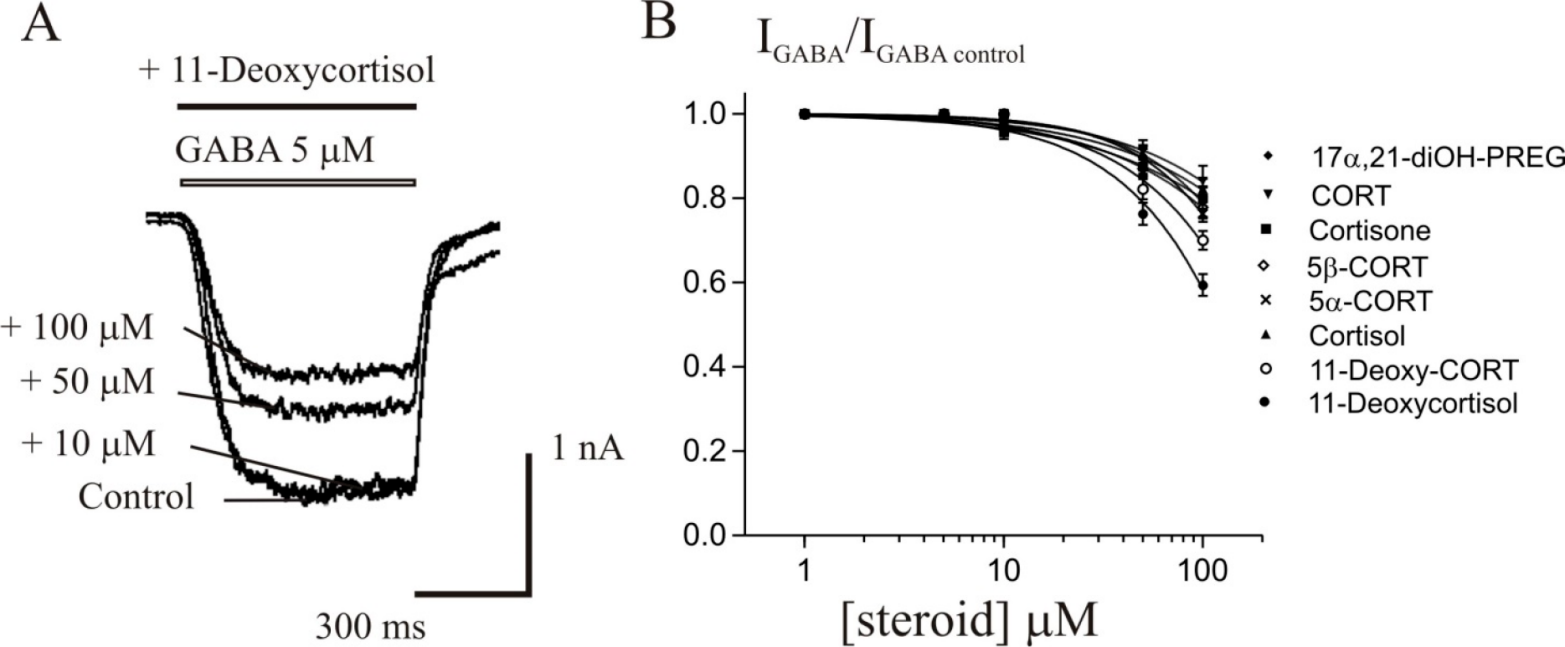
Weak inhibitory effect of 11-deoxycortisol and 11-deoxy-CORT on *I*_GABA_ in Purkinje cells. (A) Representative traces of *I*_GABA_ induced by 600 ms application of 5 μM GABA were obtained in control and in the presence of 10, 50, and 100 μM 11-deoxycortisol. (B) Concentration dependence of 17α,21-diOH-PREG, CORT, cortisone, 5β-CORT, 5α-CORT, cortisol, 11-deoxy-CORT and 11-deoxycortisol effects on the normalized peak amplitude of *I*_GABA_. *N* = 7 cells for every point.

The group of corticosteroids and their derivatives that do not contain a 3α-OH group consisted of eight substances: cortisol, cortisone, corticosterone (CORT), 11-deoxycortisol, 11-deoxycorticosterone (11-deoxy-CORT), 17α,21-dihydroxypregnenolone (17α,21-diOH-PREG), 5α-dihydrodeoxycorticosterone (5α-CORT), and 5β-dihydrodeoxycorticosterone (5β-CORT). None of these steroids potentiated the *I*_GABA_ values of Purkinje cells (Figure 4). Two of them, 11-deoxycortisol and 11-deoxy-CORT, had an inhibitory effect on the *I*_GABA_, which was noticeable only at high concentrations of steroids, 50 and 100 μM (Figure 4A). At a concentration of 100 μM, 11-deoxycortisol reduced the amplitude of the *I*_GABA_ to 58 ± 5% of the control, and 11-deoxy-CORT reduced the amplitude to 70 ± 3% of the control (*p* < 0.01, *N* = 7). The rest of the steroids from this group were inert within the range of the used concentrations (Figure 4B).

### Inhibitory Effect of Corticosteroids on *I*_Gly_ in Hippocampal Neurons

**Figure 5:**
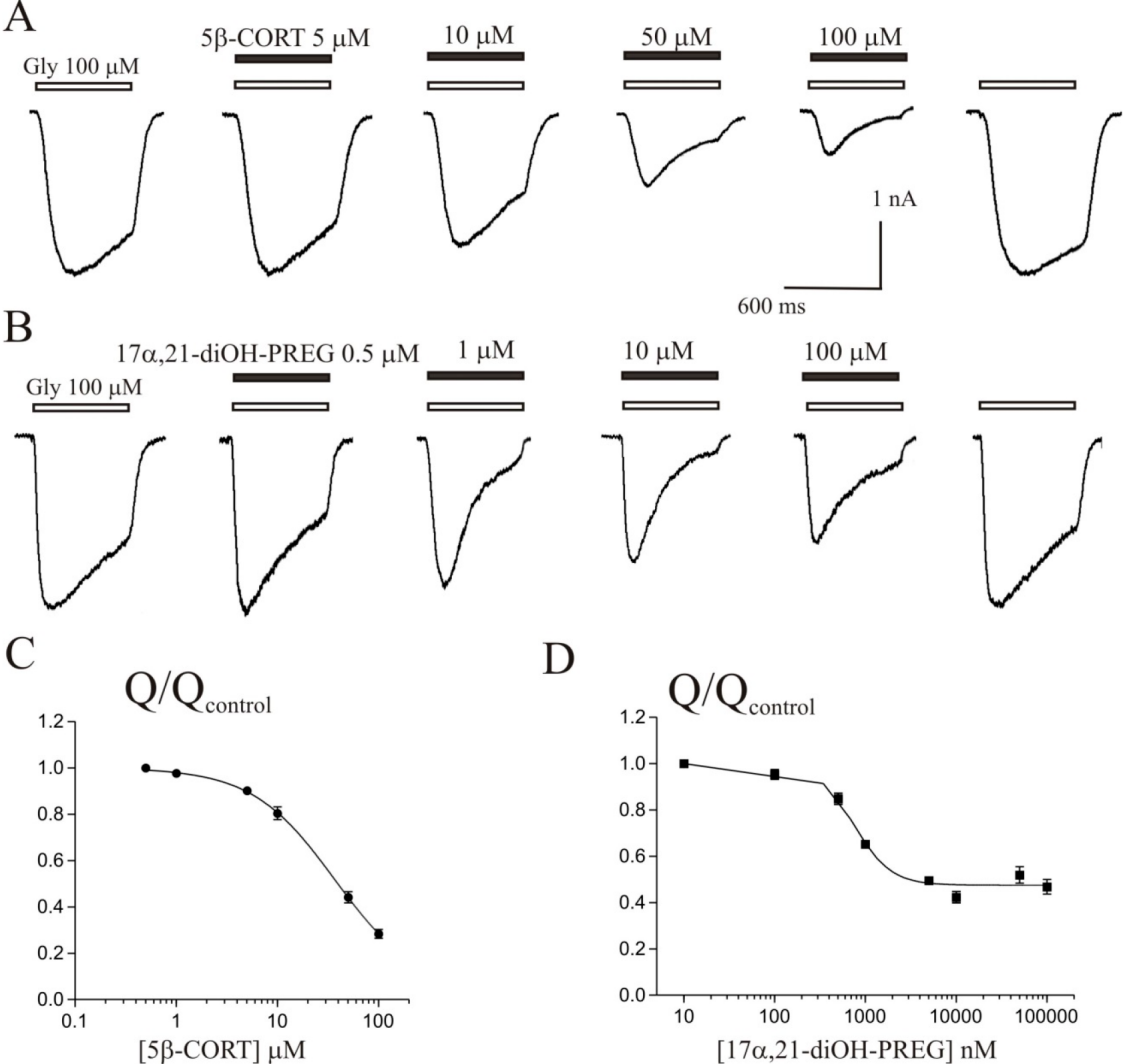
Inhibitory effect of corticosteroid derivatives on *I*_Gly_ in hippocampal neurons. (A, B) Representative traces of *I*_Gly_ induced by 600 ms application of 100 μM glycine (Gly), obtained in control and in the presence of 5, 10, 50, and 100 μM 5β-CORT (A) or 0.5, 1, 10, and 100 μM 17α,21-diOH-PREG (B). (C, D) Concentration dependence of steroid effects on the normalized charge of *I*_Gly_ (*Q*): 5β-CORT (C) and 17α,21-diOH-PREG (D). *N* = 7–8 cells for every point.

Short (600–1000 ms) application of 100 μM glycine on pyramidal neurons of rat hippocampus evoked *I*_Gly_ in which amplitude and kinetics were dependent on glycine concentration with an EC_50_ value of 90 ± 7 μM. An average value of the reversal potential of *I*_Gly_ (−9.6 ± 0.8 mV) matched well the chloride reversal potential calculated for the chloride concentrations used (−9.5 mV, not shown). All tested corticosteroids reduced *I*_Gly_ by decreasing the peak amplitude and accelerating desensitization (reducing the time constant of desensitization, τ_des_). The inhibitory effect disappeared after 2–3 applications of pure agonist. The representative traces of *I*_Gly_ from two different cells, on which the effects of 5β-CORT and 17α,21-diOH-PREG were studied, are shown in Figure 5A,B. Note that the effects of these two steroids on *I*_Gly_ are not completely identical. Both substances cause a similar effect on the peak *I*_Gly_ amplitude, which gradually decreases with an increased concentration of the steroid. However, the effect on desensitization is different on these two cells. 5β-CORT causes a gradual increase in desensitization as concentration increases, but in the case of 17α,21-diOH-PREG this gradualness is broken. The decay (desensitization) of the *I*_Gly_ gradually increases with an increase of 17α,21-diOH-PREG concentration up to 10 μM but weakens with a further increase in steroid concentration to 100 μM.

Response to glycine and its changes in the presence of 5β-CORT and 17α,21-diOH-PREG were quantified by measuring the area under the *I*_Gly_ curve. This value corresponds to the charge (*Q*) of chloride ions flowing through the chloride pore of the glycine receptor when it is activated by glycine. The value of *Q* can be calculated by the formula *Q* = *It* where *I* is the amplitude of the current, and *t* is the duration of the current. Figure 5C shows the concentration dependence of the 5β-CORT effect on the normalized *Q* of the *I*_Gly_. This steroid caused a decrease in the *Q* value with a maximum inhibition (max) of 0.96 ± 0.15, IC_50_ value of 37 ± 13 μM, and slope factor (Hill coefficient) of 1.0 ± 0.2 (*N* = 7). The inhibitory effect of 17α,21-diOH-PREG on the normalized *Q* value is shown in Figure 5D, and it has the following characteristics: max of 0.52 ± 0.01, IC_50_ value of 0.73 ± 0.06 μM, and Hill coefficient of 2.1 ± 0.4 (*N* = 8). The graph in Figure 5D shows that an increase in the steroid concentration above 10 μM does not lead to noticeable changes in the *Q* value. Meanwhile, the current records show significant, albeit multidirectional, changes in the peak amplitude and τ_des_ of *I*_Gly_ at high steroid concentrations. In our opinion, for a more detailed study of the mechanisms of the effects of steroids on *I*_Gly_, it is necessary to separately plot concentration curves for the peak amplitude and τ_des_ of *I*_Gly_.

**Figure 6:**
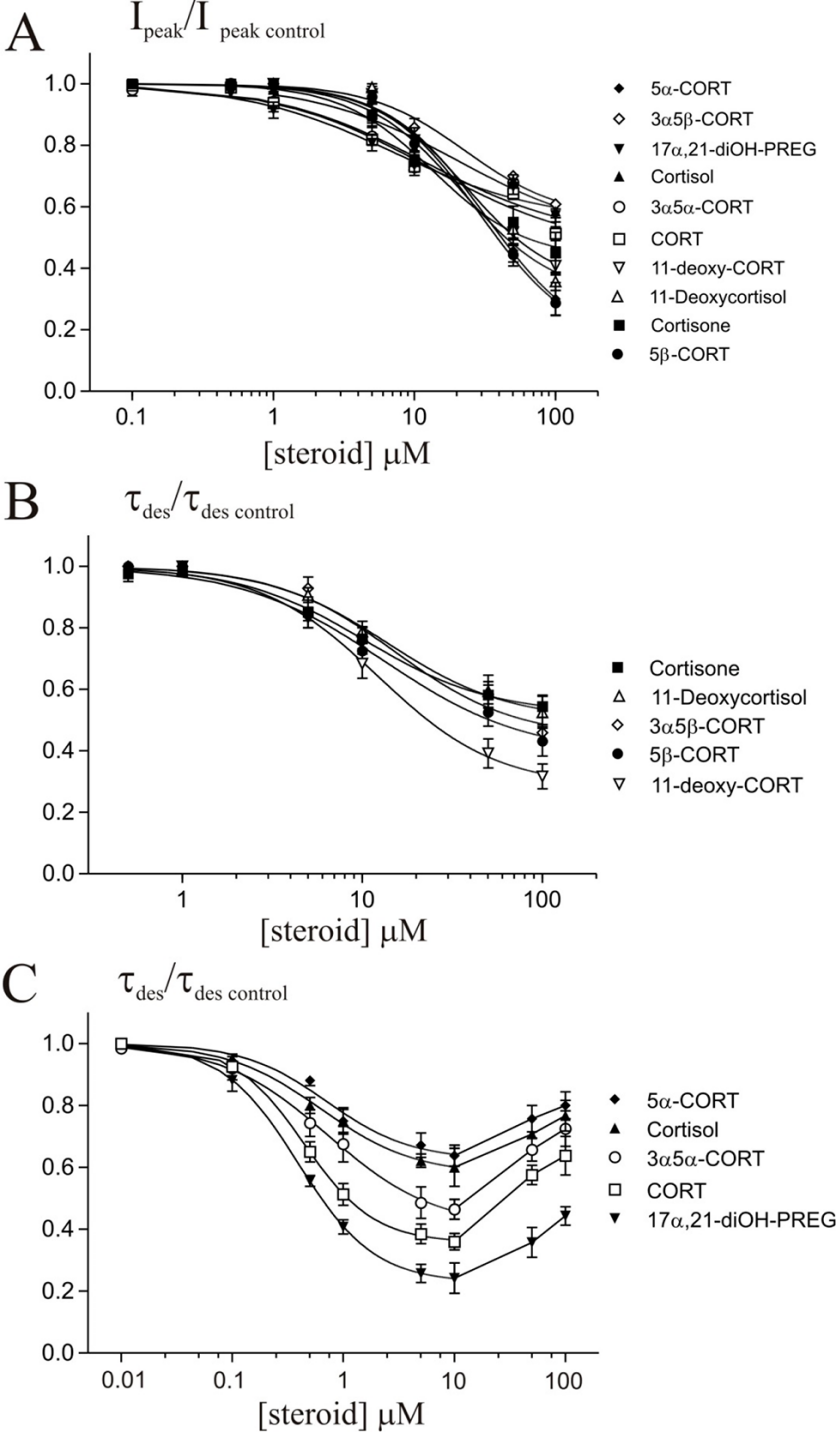
Concentration dependence of corticosteroids and their derivative effects on the normalized peak amplitude (*I*_peak_) and time constant of desensitization (τ_des_) of the *I*_Gly_. (A) Concentration dependence of ten tested steroids on the *I*_peak_ of the *I*_Gly_. (B and C) Concentration dependence of the effects of low potency (B) and high potency (C) steroids on τ_des_ of the *I*_Gly_.

Figure 6 shows the concentration dependence of the steroid effect on the normalized *I*_Gly_ peak amplitude and τ_des_. The concentration-dependence curves for the effect on the peak amplitude of *I*_Gly_ were smooth for all of the tested steroids (Figure 6A). Corticosteroids caused a decrease in the peak amplitude of the *I*_Gly_ with a threshold concentration of 10 μM, maximum inhibition (max) by 44–70%, IC_50_ values of 7.2–32 μM, and slope factor (Hill coefficient) of 0.7–1.3 (Figure 6A and Table 2, *N* = 7–8). The effect of corticosteroids on the desensitization of *I*_Gly_ varied between substances. Consequently, two types of effects could be distinguished. The first type represents effects that developed gradually with an increase in the concentration of the steroid. In particular, the relationship between the steroid concentration and τ_des_ value had a smooth appearance. This group included five steroids: cortisone, 5β-CORT, 3α5β-CORT, 11-deoxycortisol, and 11-deoxy-CORT. These compounds caused a decrease in the τ_des_ value of the *I*_Gly_ with a threshold concentration of 10 μM, maximum inhibition (max) by 48–72%, IC_50_ values of 10.3–15.2 μM, and slope factor (Hill coefficient) of 1.1–1.3 (Figure 5B and Table 2, *N* = 7–8). The second type of effect on τ_des_ was characterized by a complex dependence on the concentration of the steroid. This group also included five steroids: CORT, 5α-CORT, 3α5α-CORT, cortisol, and 17α,21-diOH-PREG. The effects of these substances on τ_des_ value reached a maximum at 5–10 μM and decreased at higher steroid concentrations. Therefore, the curve had a U-shaped form (Figure 6C). The characteristics of the effects were determined by fitting the curve to the falling phase of the curve. The analysis showed that steroids from the second group have an order of magnitude higher potency in accelerating *I*_Gly_ desensitization than steroids from the first group. These substances caused a decrease in the τ_des_ value of *I*_Gly_ with a threshold concentration of 0.1–0.5 μM, maximum inhibition (max) by 37–77%, IC_50_ values of 0.39–0.72 μM, and a slope factor (Hill coefficient) of 0.9–1.4.

**Table 2:** Values of the Maximum Inhibition Attainable (max), the Half-Maximal Inhibitory Concentration (IC_50_), and the Slope Factor (Hill Coefficient) for the Effects of Tested Steroids on the Peak Amplitude (*I*_peak_) and τ_des_ of the *I*_Gly_

	*I*_peak_			τ_des_		
compound	max	IC_50_ (μM)	Hill coefficient	max	IC_50_ (μM)	Hill coefficient
CORT	0.54 ± 0.1	12.6 ± 4.8	0.7 ± 0.1	0.64 ± 0.02	0.43 ± 0.05	1.4 ± 0.2
5α-CORT	0.86 ± 0.2	32.0 ± 12.6	1.3 ± 0.2	0.37 ± 0.04	0.71 ± 0.02	1.1 ± 0.4
3α5α-CORT	0.51 ± 0.1	11.8 ± 5.7	0.8 ± 0.2	0.59 ± 0.08	0.72 ± 0.03	0.9 ± 0.3
Cortisol	0.49 ± 0.1	20.2 ± 9.8	0.9 ± 0.2	0.42 ± 0.05	0.63 ± 0.02	1.0 ± 0.3
17α,21-diOH-PREG	0.45 ± 0.1	7.2 ± 2.6	0.8 ± 0.1	0.77 ± 0.03	0.39 ± 0.05	1.3 ± 0.2
3α5β-CORT	0.44 ± 0.1	23.0 ± 9.6	1.3 ± 0.3	0.56 ± 0.08	15.2 ± 6.2	1.3 ± 0.5
5β-CORT	0.85 ± 0.3	29.3 ± 9.9	1.3 ± 0.3	0.61 ± 0.09	13.1 ± 5.8	1.1 ± 0.4
Cortisone	0.57 ± 0.1	13.6 ± 5.5	1.3 ± 0.5	0.48 ± 0.06	10.3 ± 3.4	1.2 ± 0.5
11-Deoxycortisol	0.69 ± 0.2	21.4 ± 7.6	1.3 ± 0.3	0.50 ± 0.08	13.8 ± 6.3	1.3 ± 0.5
11-deoxy-CORT	0.67 ± 0.2	22.9 ± 9.4	1.3 ± 0.4	0.72 ± 0.08	12.2 ± 3.7	1.3 ± 0.2

The literature shows that the effect of neurosteroids on the GABA response can change from inhibitory to potentiating with a decrease in GABA concentration.^27^ According to the literature, no such effect has been evaluated for the glycine-induced current. Therefore, we have tested the effect of 1 and 50 μM 11-deoxy-CORT on *I*_Gly_ with a glycine concentration of 25 μM (EC_10_). No reversal of the inhibitory effect into a potentiating one was identified; 11-deoxy-CORT (1 μM) did not affect the *I*_Gly_ and 11-deoxy-CORT at 50 μM concentration caused a decrease in the peak amplitude of the *I*_Gly_ by 45 ± 7% (*N* = 6, *P* < 0.001) (not shown).

## Discussion

Our research presents the effect of three corticosteroids, namely, cortisol, corticosterone, and cortisone, and seven of their derivatives on the *I*_GABA_ of isolated Purkinje neurons and the *I*_Gly_ of pyramidal neurons in the rat hippocampus. Inhibitory GABA neurotransmission is thought to participate in the mobilization mechanisms of the brain under acute stress.^7^ Under exposure to acute stressors, allopregnanolone and THDOC concentrations can be increased enough to influence GABA inhibition,^28^ which provides neuroprotective and anticonvulsant effects.^7^ THDOC is a 3α5α-derivative of corticosterone and, as shown by many authors,^11,29,30^ is able to enhance *I*_GABA_ at nanomolar concentrations (30–100 nM) in various model systems. Protein kinase C has been shown to be involved in the mechanisms of this potentiation^31^ In our experiments, we studied two 11-hydroxy analogues of THDOC, namely, 3α5α-CORT and 3α5β-CORT. They both caused an increase in *I*_GABA_ in isolated Purkinje cells with EC_50_ values of 23 μM and 18 μM, correspondingly. These high EC_50_ values for 11β-hydroxylated derivatives compared to that for THDOC indicate that hydroxylation reduces the ability of steroids to enhance GABA neurotransmission. Although we have shown that the presence of the 11β-hydroxyl group diminished the ability to potentiate *I*_GABA,_ it should be noted that 11α-hydroxylated allopregnanolone and 11α-hydroxylated pregnanolone have been shown to be essentially active as potentiators.^32,33^ The remaining eight substances do not have a 3α-hydroxyl group and do not potentiate *I*_GABA_. Literature data on the role of the 3α-hydroxyl group of the steroid skeleton in GABA_A_R potentiation are ambiguous. It has been shown that steroids, both containing and not containing the 3α-hydroxyl group, can potentiate GABA_A_R.^26,34^

This study aimed to evaluate corticosteroids and their metabolites as potential selective modulators of *I*_Gly_. The identification of selective steroidal modulators of GlyRs would lead to novel approaches to the development of steroidal neurotherapeutics. Indeed, in our study, we have shown that GlyRs are a more sensitive target for corticosteroids than GABA_A_Rs. Five of ten substances inhibited the *I*_Gly_ of pyramidal neurons with high efficiency and potency. This group included: corticosterone (CORT), cortisol, 5α-CORT, 3α5α-CORT, and 17α,21-diOH-PREG. All of them reduced *I*_Gly_, decreasing peak amplitude and accelerating desensitization. It is important to note that the effect of accelerating desensitization required a 10-fold lower concentration of drugs (IC_50_ of 0.39–0.72 μM) than the effect of reducing the peak amplitude of *I*_Gly_ (IC_50_ of 7.2–32 μM). Such a different regulation of these two parameters by steroids suggests the existence of two independent mechanisms of their action on GlyRs, one of which regulates the peak amplitude, and the second–the desensitization process. This assumption was confirmed in our previous publications,^24^ where we showed that at high concentrations of glycine (500 μM), the effect of neurosteroids on the peak amplitude of *I*_Gly_ disappears, but the effect on desensitization does not change. In our opinion, the binding of steroids with a desensitization gate in a pore of the chloride channel^35,36^ is the simplest explanation of the acceleration of desensitization. However, we do not deny the possibility of another mechanism of this effect, namely, the slow block involved, for example, in the effect of accelerating the decay of NMDA current under the influence of neurosteroids.^37^

The dose–effect curves for the acceleration of desensitization were U-shaped in the presence of these five steroids. This complex form of dose–response may indicate that the process activated by high concentrations of steroids inhibits the process that is sensitive to low concentrations of them. At present, only limited literature is available about the molecular basis of the action of steroids at GlyRs. To date, our knowledge of NS binding sites on GlyR is limited to the description of five identical α–α inter-subunit sites at the homopentamer GlyR-α3.^38^ However, it is hoped that multiple NS sites, both inter-subunit and intra-subunit, will be described on GlyR, as is the case for GABA_A_R.^39^ It can be assumed that some of these sites are responsible for desensitization and can interact with each other. The results of this work generally coincide with our previous results obtained on the same model with other steroids: pregnanes,^26^ androstanes, and androstenes.^25^ With those steroids, we reported the same tendency, i.e., higher potency of the substances to accelerate desensitization than to reduce the peak amplitude of *I*_Gly_.

Interestingly, in the literature, we have not found any indications of the ability of NSs to accelerate the desensitization of *I*_Gly_. The published studies of the action of steroids on *I*_Gly_ were performed on recombinant GlyRs expressed in frog oocytes,^23,40^ a chicken spinal neuron culture,^21^ and a rat hippocampal and spinal neuron culture.^19,20^ In all of the mentioned models, the authors observed a change in the *I*_Gly_ peak amplitude under the influence of NSs. A comparison of our data with those of other authors allows us to conclude that the use of such a model as acutely isolated pyramidal neurons of the hippocampus with a system of fast application provides a unique opportunity to study the processes of GlyR desensitization.

However, not all corticosteroids in our experiments had a high potency for GlyR inhibition. Five substances (cortisone, 11-deoxycortisol, 11-deoxy-CORT, 5β-CORT, and 3α5β-CORT) accelerated the desensitization of *I*_Gly_ at much higher concentrations (the IC_50_ values of 10.3–15.2 μM). In contrast to the first group of steroids with high activity, in this group, the dose–response curve had a smooth appearance, which indicates the interaction of steroids with one type of low-affinity site on GlyR.

An important aspect of our study is the physiological relevance of the active doses of the studied corticosteroids. Not surprisingly, cortisol has the highest physiological concentration of the three stress hormones that we tested (cortisol, cortisone, and corticosterone). The concentration of cortisol depends on gender, age, and pubertal stage. Moreover, it increases gradually from the first year of life span (425 nM) toward adulthood (up to 616 nM).^41^ A high level of cortisol (1326 nM) was observed in women taking oral contraceptives.^41^ The course of some neuropsychiatric diseases was shown to be accompanied by an increase in cortisol level, for example, Alzheimer’s disease (647 nM),^42^ Cushing’s syndrome (2735 nM),^43^ and anxiety (801 nM).^44^ In our experiments, the three most active substances accelerated the desensitization of *I*_Gly_ with the following IC_50_ values: 17α,21-diOH-PREG at 390 nM, corticosterone at 430 nM, and cortisol at 630 nM. We conclude that these IC_50_ values correspond to the physiological concentration of cortisol in the serum of adult humans. It should also noted that corticosteroids are widely used to treat a variety of illnesses, and in these cases, their serum concentrations can significantly exceed normal physiological values.^45^

Taken together, our work demonstrates the ability of stress hormones, corticosterone and cortisol, to suppress GlyR functions with nanomolar concentrations. Along with GABA_A_Rs, GlyRs perform an inhibitory function in the nervous system and are involved in multiple essential physiological processes such as motor coordination, respiratory rhythms, pain transmission, sensory processing, and neurodevelopment.^46^ Consequently, increased desensitization of GlyRs by cortisol and corticosterone can lead to a decrease in inhibition in the brain and a change in physiological status. Under chronic stress, reduced inhibition in the brain is observed, which is explained by the depletion of neurosteroids, activators of GABA_A_Rs.^7^ Our results indicate the existence of another possible mechanism for this pathology, namely, increased GlyRs desensitization.

Finally, our study has demonstrated that corticosterone (CORT), cortisol, 5α-CORT, 3α5α-CORT, and 17α,21-diOH-PREG act as selective modulators of GlyRs. These findings offer novel avenues for structure–activity relationship studies targeting novel steroidal neurotherapeutics.

## Materials and Methods

### Cell Preparation

All experiments were conducted per the requirements of the Ministry of Public Health of the Russian Federation and were consistent with the EU directive for the Use of Experimental Animals of the European Community. The cells were isolated from rat brain slices as described in detail elsewhere.^47^ Briefly, the slices (200–500 μm) of Wistar rat (11–14 days of age) hippocampus or cerebellum were incubated at room temperature for at least 2 h in a solution containing the following components (in mM): 124 NaCl, 3 KCl, 2 CaCl_2_, 2 MgSO_4_, 25 NaHCO_3_, 1.3 NaH_2_PO_4_, and 10 d-glucose, pH 7.4. The saline was continuously stirred and bubbled with carbogen (95% O_2_ + 5% CO_2_). Single pyramidal neurons from the hippocampal CA3 area or Purkinje cells from sagittal slices of the cerebellum were isolated by a vibrating fused glass pipet with a spherical tip.

### Current Recordings

Glycine-activated currents (*I*_Gly_) and GABA-activated currents (*I*_GABA_) in isolated neurons were induced by a step application of an agonist for 600–1000 ms with 30–40 s intervals through a glass capillary, 0.1 mm in diameter, which could be rapidly displaced laterally. Transmembrane currents were recorded using a conventional patch-clamp technique in the whole-cell configuration. Patch-clamp electrodes had a tip resistance of ∼2 MΩ. The solution in the recording pipet contained the following (in mM): 40 CsF, 100 CsCl, 0.5 CaCl_2_, 5 EGTA, 3 MgCl_2_, 4 NaATP, 5 HEPES, pH 7.3. The composition of the extracellular solution was as follows (in mM): 140 NaCl, 3 KCl, 3 CaCl_2_, 3 MgCl_2_, 10 d-glucose, 10 HEPES hemisodium, pH 7.4. The speed of perfusion was 0.6 mL/min. Recording of the currents was performed using an EPC7 patch-clamp amplifier (HEKA Elektronik, Germany). The holding potential was maintained at −70 mV. Transmembrane currents were filtered at 3 kHz, stored, and analyzed with IBM-PC computer, using homemade software.

### Reagents

All of the drugs used for intracellular and extracellular solutions were purchased from Sigma-Aldrich (USA). Corticosteroid compounds were purchased as follows: cortisol (CAS 50-23-7, catalogue ID ENAH97EDF0AB, Merck Life Science spol. s r.o., Prague Czech Republic), corticosterone (CAS 50-22-6, catalogue ID 27840, Merck Life Science spol. s r.o., Prague Czech Republic), cortisone (CAS 53-06-5, catalogue ID C2755, Merck Life Science spol. s r.o., Prague Czech Republic), 17α,21-dihydroxypregnenolone (CAS 1167-48-2, catalogue ID D454585, Toronto Research Chemicals, Toronto, Canada), 5α-dihydrodeoxycorticosterone (CAS 298-36-2, catalogue ID D448790, Toronto Research Chemicals, Toronto, Canada), 5β-dihydrodeoxycorticosterone (CAS 303-01-5, catalogue ID H941558, Toronto Research Chemicals, Toronto, Canada), 11-deoxycorticosterone (CAS 64-85-7, catalogue ID D6875, Merck Life Science spol. s r.o., Prague Czech Republic), 11-deoxycortisol (CAS 152-58-9, catalogue ID PHR2158, Merck Life Science spol. s r.o., Prague Czech Republic), allotetrahydrocorticosterone (CAS 567-03-3, catalogue ID T293270, Toronto Research Chemicals, Toronto, Canada), and tetrahydrocorticosterone (CAS 68-42-8, catalogue ID T293160, Toronto Research Chemicals, Toronto, Canada). Steroids were dissolved in DMSO as 10–100 mM stock solutions, which were aliquoted and stored at −20 °C. The stock solution was diluted before the experiments in external bath saline to the final concentrations. The drug solutions remained stable during the experiments. The final concentration of DMSO was 0.1% at maximum, and DMSO itself at this concentration had no effect on the *I*_Gly_ or *I*_GABA_.

### Data Analysis

Statistical analysis was performed with Prism Graphpad software. All comparisons were made with unpaired (Figures 2, **4**, **5**, and **6**) or paired (Figure 3) Student’s *t*-test at a significance level of *p* = 0.05. In results descriptions, the mean and standard error of mean (SEM) are specified. *N* = 6–8 cells from 3 animals for every average value. The IC_50_ values for steroid inhibition of the *I*_Gly_ and *I*_GABA_ were determined using the equation: *Y* = 1 – [max/(1 + (IC_50_/*C*)^*n*^)], where max is the maximum inhibition attainable, *C* is the concentration of steroid, IC_50_ is the half-maximal inhibitory concentration, and *n* is the slope factor (Hill coefficient). The EC_50_ value for steroid potentiation of the *I*_GABA_ was determined using the equation: *Y* = Bottom + (Top – Bottom)/[1 + (EC_50_/*C*)^*n*^], where Bottom and Top are current amplitudes measured in the control solution and in the presence of steroid, respectively, *C* is the concentration of steroid, EC_50_ is the half-maximal stimulating concentration, and *n* is the slope factor (Hill coefficient).
